# Negligible Risk for Epidemics after Geophysical Disasters

**DOI:** 10.3201/eid1204.051569

**Published:** 2006-04

**Authors:** Nathalie Floret, Jean-François Viel, Frédéric Mauny, Bruno Hoen, Renaud Piarroux

**Affiliations:** * University Hospital of Besançon¸ Besançon, France;; † University of Franche-Comté, Besançon, France

**Keywords:** Earthquake, volcano eruption, tidal wave, tsunami, epidemics

## Abstract

After geophysical disasters (i.e., earthquakes, volcanic eruptions, tsunamis), media reports almost always stress the risk for epidemics; whether this risk is genuine has been debated. We analyzed the medical literature and data from humanitarian agencies and the World Health Organization from 1985 to 2004. Of >600 geophysical disasters recorded, we found only 3 reported outbreaks related to these disasters: 1 of measles after the eruption of Pinatubo in Philippines, 1 of coccidioidomycosis after an earthquake in California, and 1 of *Plasmodium vivax* malaria in Costa Rica related to an earthquake and heavy rainfall. Even though the humanitarian response may play a role in preventing epidemics, our results lend support to the epidemiologic evidence that short-term risk for epidemics after a geophysical disaster is very low.

## Abstract 2

Short-term risk for epidemics after geophysical disasters is very low.

Natural disasters are defined as "a disruption of human ecology which exceeds the community's capacity to adjust, so that outside assistance is needed" (1). Their classifications are geophysical (earthquakes, volcanic eruptions, tsunamis), hydrometeorologic (floods and wind storms), and geomorphologic (landslides). When covering these events, media outlets almost always mention the risk for epidemics that could raise the death toll well above an already staggering number of victims. According to the Centers for Disease Control and Prevention (CDC), an epidemic is the occurrence of more cases of disease than expected in a given area or among a specific group of persons over a particular period of time. For many, the word epidemic is associated with large numbers of deaths and poor living conditions, such as those that sometimes occur in refugee camps (2). The term outbreak is synonymous with epidemic and is sometimes preferred because it may not evoke the sensationalism associated with the word epidemic.

In addition to the media, other outlets draw attention to the risk for epidemics. In a letter published 3 weeks after the earthquake in Bam, Iran, in December 2004, the World Health Organization (WHO) warned that potential outbreaks of cholera, typhoid fever, malaria, and leishmaniasis were a major concern (3). WHO also issued a warning about the risk for epidemics that could develop after the 2004 tsunami: "There is an immediate INCREASED RISK of waterborne diseases, i.e., cholera, typhoid fever, shigellosis and hepatitis A and E…. Outbreaks of these diseases could occur at any moment" (4). The high risk for epidemics in areas affected by the tsunami was also pointed out by several papers published during the weeks after the disaster (5,6). Responding to WHO announcements, humanitarian agencies invested effort, time, personnel, and money in gearing up for potential epidemics, and considerable stocks of antimicrobial drugs, rehydration fluids for cholera patients, and vaccines were sent to the field.

However, not all experts support these alarming predictions. Some experts hold that disasters do not usually result in disease outbreaks but may increase disease transmission under certain circumstances (e.g., fecal contamination of water, spread of respiratory diseases in evacuation camps) (7). A similar point of view was published by VanRooyen and Leaning (8) and by de Ville de Goyet (9), who spoke of the myths propagated after disasters, some of which lead to an overestimation of the risk for epidemics.

No article has systematically reviewed published reports dealing with epidemics after geophysical disasters. The role played by outbreaks of infectious diseases in causing illness after geophysical disasters must be identified so that priorities can be defined and resources can be appropriately allocated. A systematic review of medical literature could help answer the question, "Is the risk for epidemics high after a geophysical disaster?" Consequently, we analyzed medical literature of the past 20 years and data provided by several websites and databases that compile outbreak alert messages and situation reports after disasters.

## Materials and Methods

### Literature Review

We screened Medline for articles that described outbreaks and epidemics, in both English and French, published from January 1985 through December 2004. We used the following search terms: (natural disaster* OR seism* OR earthquake* OR volcano* OR tsunami*) AND (infectious disease* OR communicable disease* OR epidemic* OR outbreak* OR vector-borne disease* OR arboviruses OR cholera OR malaria OR dengue OR West Nile virus OR Rift Valley fever OR hepatitis OR leptospirosis OR typhoid fever OR measles OR shigellosis OR scrub typhus OR plague OR diarrhea). We first selected all articles related to a specific earthquake, volcanic eruption, or tsunami, and then we examined them for any quantitative data on at least 1 infectious disease.

### Screening Databases on the Internet

Data on epidemics and geophysical disasters were collected from the following databases: Emergency Disasters Data Base (Em-Dat, http://www.em-dat.net), WHO websites (http://www.who.int/), Disease Outbreak News (http://www.who.int/csr/don/en/), Centers for Disease Control and Prevention (http://www.cdc.gov/), Morbidity and Mortality Weekly Report (http://www.cdc.gov/mmwr/), and the Pan America Health Organization (http://www.paho.org). Research focused on events that occurred from January 1985 through December 2004. For disasters that were responsible for >100 deaths, we systematically screened reports of humanitarian agencies available on Reliefweb (http://www.reliefweb.int/).

## Results

### Literature Review

Although we found 233 articles in the Medline database related to our query, only 18 (7.7%) actually reported on infectious disease data collected after geophysical disasters. Common respiratory tract infections and diarrhea were the most frequently reported diseases. After the Bam earthquake in December 2003, a survey of 75,586 displaced persons described the main health problems encountered (10). Respiratory tract infections (mainly upper respiratory tract infections) were most frequently encountered; 11,320 cases were seen in the 10 weeks after the disaster. Researchers attributed the high number of respiratory infections to the freezing winter nights. Diarrhea was commonly diagnosed (1,224 cases with 174 cases of bloody diarrhea), but *Vibrio cholerae* infection was not observed. Similar findings were reported after the Chi-Chi earthquake in Taiwan in September 1999. An epidemiologic survey conducted in shelters showed that acute respiratory infections and acute gastroenteritis were the most common illnesses reported (11). They increased during the first 4 weeks, were significantly higher than those in unaffected neighboring counties, and then declined to baseline levels afterwards. An increase in gastrointestinal and respiratory infections also followed the 2001 earthquakes in El Salvador (12). Woersching and Snyder. conducted a 32-question survey in 100 households (594 persons) severely affected by the earthquakes. These researchers found that 30% of households assessed experienced >1 case of upper respiratory infection, and 22% experienced >1 case of diarrheal disease. This study also showed a high frequency of skin infections (31% of households). In a figure, authors reported 6 cases of cholera but did not state whether the diagnosis was biologically confirmed (no cases of cholera were officially identified in El Salvador in 2001) (13). An increase in respiratory and intestinal tract infections was also reported after the eruption of the Cerro Negro volcano in Nicaragua in April 1992, although this increase was not declared an epidemic (14). An assessment of the health consequences of the disaster showed that acute diarrhea was 6 times more frequent after the eruption, and medical consultations for acute respiratory disease were 3.6 times more frequent than before.

Two studies were performed to assess medical records of inpatients hospitalized during the first 15 days after the Hanshin-Awaji earthquake in January 1995 (15,16). Among infectious diseases, pneumonia was the most frequent illness diagnosed in inpatients (13%–21% according to the 2 surveys). An increased number of inpatients were also recorded in Papua New Guinea after the tsunami in July 1998 (17). However, no outbreak of communicable disease occurred.

A few studies investigated the prevalence of some pathogens in persons living in shelters. After the earthquake in Turkey in August 1999, an analysis of 1,468 stool cultures taken from persons with diarrhea showed 92% negative results; the most frequently isolated pathogens were *Shigella* spp. (4.9%). Phenotypic and genotypic comparisons of strains showed no cloning among the *Shigella* strains (18). Another study was conducted to determine the influence of the earthquake on patient admittance to the outpatient dermatology clinic. The incidence of skin infections was higher after the earthquake than it was in the same period 1 year later (19). A third study was performed to assess the prevalence of hepatitis A and E among children living in camps in northwestern Turkey (20). Hepatitis A and E virus seroprevalence was higher in the camps around Golyaka (68.8% and 17.2%, respectively) than in camps around Düzce (44.4% and 4.7%). The authors suggested that these differences were possibly related to delays in obtaining toilet facilities and piped water. After the earthquake in Colombia in January 1999, a parasitologic survey was performed in transitory housing camps from January 2000 to July 2001 (21). A high prevalence of *Giardia* spp. (60%) was found in stool specimens of 217 randomly selected children, and this prevalence was significantly associated with the use of communal toilets instead of individual toilets and with drinking municipal water instead of water from individual tanks. The authors also stated that no outbreak of diarrhea, dengue fever, or malaria had occurred.

Only 3 articles reported outbreaks after a geophysical disaster. An outbreak of malaria (due to *Plasmodium vivax*) was reported after an earthquake in April 1991 in Costa Rica (22). From June 1991 through May 1992, a total of 3,597 cases were recorded, compared to 549 and 681 cases for the same period during the 2 preceding years. Even though heavy rainfall occurred in August 1991, authors suggested that the earthquake may have played a role. An outbreak of coccidioidomycosis was described after the 1994 earthquake in Northridge, California. The attack rate reached 30 cases per 100,000 inhabitants. According to the authors, being in a dust cloud and the amount of time spent in a dust cloud were associated with an increased risk of diagnosis (23). An outbreak of measles occurred after the eruptions of Mt. Pinatubo in June 1991. By August, many children of the Aeta tribe, who usually lived in isolation on the slopes of Pinatubo, had died in evacuation centers. The death toll reached 349 in the first 12 weeks, accounting for a death rate of 26/10,000 by the seventh week after the eruption (24–26). Deaths were caused by measles (31%), diarrhea (29%), and respiratory infections (22%). Living conditions were extremely difficult in camps: tents provided only minimal shelter from the elements, and evacuees experienced extremely hot days and cold, damp nights (26). Malnutrition and lack of basic sanitation also contributed to high death rates among children (24).

### Database Research

From 1985 to 2004, 516 earthquakes, 89 volcano eruptions, and 16 tidal waves or tsunamis (including the December 2004 tsunami) were identified in the Em-Dat database. Sixty-three of these geophysical disasters were responsible for >100 deaths each, and 26 of them were responsible for >1,000 deaths (Table). Most of them (55 of 63) were reported on the ReliefWeb site. However, only 21 descriptions included medical data that covered at least the 3-month period after the disaster. Only 1 outbreak was reported: 19 cases of Crimean-Congo hemorrhagic fever, including 12 fatal cases, occurring in mid-March 1998 in a village in the district of Rustaq, Afghanistan, where an earthquake had occurred in February 1998. This outbreak was not caused by the earthquake but was detected because of epidemiologic surveillance that was implemented after the earthquake. No outbreak was reported after the other disasters, even in reports published up to 3 months after the events.

**Table:** Infectious diseases and outbreaks after major disasters (>1,000 deaths), 1985–2004

Date	Type of disaster	Location	No. deaths	Infectious diseases and outbreaks*
September 1985	Earthquake	Mexico	9,500	No report
November 1985	Volcano eruption	Colombia	21,800	Giardiasis, no outbreak
August 1986	Volcano eruption	Cameroon	1,746	No report
October 1986	Earthquake	El Salvador	1,100	No report
March 1987	Earthquake	Ecuador	5,000	No report
December 1988	Earthquake	Armenia	25,000	No report
June 1990	Earthquake	Iran	26,796	No report
July 1990	Earthquake	Philippines	2,412	No report
October 1991	Earthquake	India	20,005	No report
December 1992	Earthquake	Indonesia	2,500	No report
September 1993	Earthquake	India	9,748	No report
January 1995	Earthquake	Japan	5,297	Pneumonia
May 1995	Earthquake	Russia	1,989	No report
February 1997	Earthquake	Iran	1,728	No report
May 1997	Earthquake	Iran	1,100	No report
February 1998	Earthquake	Afghanistan	1,000	No report
May 1998	Earthquake	Afghanistan	4,700	No report
July 1998	Tsunami	Papua New Guinea	2,182	No outbreak
January 1999	Earthquake	Colombia	1,186	No report
August 1999	Earthquake	Turkey	17,127	Diarrhea, hepatitis A and E, skin infections, no outbreak
September 1999	Earthquake	Taiwan	2,264	Diarrhea, RTI, no outbreak
January 2001	Earthquake	India	1,500	No report
March 2002	Earthquake	Afghanistan	2,323	No report
May 2003	Earthquake	Algeria	2,266	No report
December 2003	Earthquake	Iran	40,000	Diarrhea, RTI, no outbreak
December 2004	Tsunami	Bay of Bengal	>200,000	No outbreak

*RTI, respiratory tract infection.

Among alert messages reported on the WHO outbreak news website, >300 concerned new outbreaks detected from 1997 to 2004 (we could not access previous WHO archives), and 90 of these concerned cholera outbreaks. We also found 779 epidemics reported in Em-Dat from 1985 to 2004. However, only 1 outbreak (of Crimean-Congo hemorrhagic fever [previously mentioned]) occurred in an area affected by a recent geophysical disaster.

## Discussion

Although >600 geophysical disasters were recorded in the 20-year period we studied, we found no report in the medical literature in which major epidemics occurred in their wake. Only 2 outbreaks, one of *Coccidioides immitis* infection and the other of measles, could clearly be related to a preceding disaster (23–26). Since this result is at variance with the fact that iterative warning messages are broadcast after each disaster, we enlarged our search of the past 20 years by checking for alert messages from various institutional disease control databases and by screening reports available on Reliefweb.

The lack of reported epidemics in all the sources we analyzed begs an essential question: If epidemics can be expected to occur after a geophysical disaster, why are they almost never detected or reported? In general, epidemiologic studies are rarely conducted after disasters, and when they are, their methods are open to criticism. Most investigations only use cross-sectional survey methods without any reference to baseline status or control areas (27). In remote, rural areas of developing countries and in areas affected by war, surveillance systems are often not functioning, and an epidemic may go unnoticed. In addition, medical humanitarian agencies mainly focus on short-term assistance to affected persons, and most volunteers and experts usually leave the area within 3 months (1). At that time, basic sanitation facilities and access to basic hygiene may still be unavailable because of economic consequences of the disaster, and some affected victims may have to stay in camps and shelters for prolonged periods. Given the flaws of epidemiologic surveys described above, the hypothesis that unreported outbreaks occur a considerable time after the onset of a disaster must be examined. However, for some diseases, such as cholera, meningitis, and dengue, a large-scale outbreak would likely be detected by local health authorities or by humanitarian agencies working after the emergency phase. In that case, WHO would be notified or a field report would be made, even though the outbreak might not be reported in a medical journal.

Many arguments are usually presented to show that a geophysical disaster is a high-risk situation for epidemics. First, water and sanitation systems may be destroyed during the disaster, increasing the risk for outbreaks of waterborne diseases. However, natural disasters do not import diseases, and even in areas where a given disease is endemic, the worst-case scenario does not always occur (9). Cholera is endemic around the Bay of Bengal, but cases of cholera are not constantly diagnosed in each village around the bay. Even if brackish water in the estuaries is an environmental reservoir for *V. cholerae*, toxigenic bacteria do not necessarily spread from them, should a tsunami occur. Many ecologic, sociologic, and seasonal factors are involved in the emergence of *V. cholerae*, and these factors rarely converge (28). Another surprising assertion is that tsunamis increase water sources for mosquitoes and therefore enhance the risk for vectorborne disease outbreaks. Water is an essential component of the mosquito environment. The characteristics of the water habitat, whether it is running or standing, clean or polluted, fresh or brackish, shaded or sunlit, permanent or intermittent, are the predominant factors determining which species of mosquito breed in it. Transient, polluted salt water generated by a tsunami will not sustain most species involved in transmission of dengue fever and malaria (29).

Second, natural disasters arguably lead to population displacement, formation of camps, overcrowding, and therefore, propitious circumstances for an epidemic. Settlements for victims of natural disasters, however, are not synonymous with refugee camps created to cope with complex emergencies (e.g., war, oppression, famine). In such complex emergencies, refugees may live for a long time in overcrowded conditions with a poor water supply and bad sanitary facilities. Usually refugees have been malnourished for weeks or even months before they reach the camps. Conditions like this in Goma, Zaire, produced epidemics of cholera, shigellosis, and meningitis, which caused thousands of deaths (30). For natural disasters, the shock is short-term, and communities can cope with problems more easily; predisaster health and nutrition status are better than in complex emergencies. The camps are often much smaller, which limits the spread of pathogens; access to food, safe water, and sanitary facilities is usually better; and most people stay only a few days or weeks. Nevertheless, crowded conditions and, in some cases, cold weather, favor the transmission of airborne diseases. The first response in preventing an outbreak of respiratory disease is to provide adequate shelter as soon as possible to injured persons and to prevent overcrowding. In our study, however, measles outbreaks were far less frequent than expected. Early implementation of immunization campaigns probably has a protective effect, and vaccination is recommended each time nonimmunized populations are moved to camps. Vaccination against influenza is not recommended even though it is a highly contagious disease and has a shorter incubation period than measles. Surprisingly, despite the lack of immunization campaigns we observed in our study, we never found any report of an influenza epidemic whose spread was aided by a preceding geophysical disaster.

The "fact" that dead bodies are a potential cause of epidemics after a disaster is also almost always broadcast after major disasters. This "fact" is a myth, and depriving survivors of appropriate burial ceremonies for their relatives may administer yet another blow to already injured or weakened persons (9,31,32). The only situation in which handling corpses is a risk is during epidemics of infectious diseases such as cholera. Even in these situations, no reason exists to totally deprive families from honoring their dead if they follow certain precautions (33).

Our results, in line with those of Noji and de Ville de Goyet, lend support to the epidemiologic evidence that no high, short-term risk for epidemics follows a geophysical disaster. While most medical topics are usually discussed in small task groups of highly specialized experts, the debate about risk for epidemics after natural disasters is usually conducted by the mass media. The news industry is prone to emphasizing more dramatic and simplistic messages, and unjustified warnings will likely continue to be spread on the basis of an approximate assessment of risks. To respond more effectively to the needs of victims of natural disasters, the public, mass media, humanitarian organizations, and policymakers must be accurately informed regarding what actions are effective and what actions are futile.

## References

[1] Lechat MF. The epidemiology of health effects of disasters. Epidemiol Rev. 1990;12:192–8. 2286219 10.1093/oxfordjournals.epirev.a036053

[2] Goma Epidemiologic Group. Public health impact of Rwandan refugee crisis: what happened in Goma, Zaire, in July 1994. Lancet. 1995;345:339–44. 10.1016/S0140-6736(95)90338-0 7646638

[3] Zarocostas J. WHO praises Bam response but warns of disease. Lancet. 2004;363:218. 10.1016/S0140-6736(03)15373-1 14746316

[4] World Health Organization. South Asia tsunami situation report 4 [monograph on the Internet]. 2005 Jan 2 [cited 2006 Feb 15]. Available from http://www.who.int/hac/crises/international/asia_tsunami/sitrep/04/en/index.html

[5] Moszynski P. Disease threatens millions in wake of tsunami. BMJ. 2005;330:59. 10.1136/bmj.330.7482.59 15637356 PMC543858

[6] Vogel G. Using scientific assessments to stave off epidemics. Science. 2005;307:345. 10.1126/science.307.5708.345 15661989

[7] Noji EK. Indian Ocean tsunami. Public health issues in disasters. Crit Care Med. 2005;33(Suppl):S29–33. 10.1097/01.CCM.0000151064.98207.9C 15640676

[8] VanRooyen M, Leaning J. After the tsunami—facing the public health challenges. N Engl J Med. 2005;352:435–8. 10.1056/NEJMp058013 15689579

[9] de Ville de Goyet C. Stop propagating disaster myths. Lancet. 2000;356:762–4. 10.1016/S0140-6736(00)02642-8 11085709

[10] Akbari ME, Farshad AA, Asadi-Lari M. The devastation of Bam: an overview of health issues 1 month after the earthquake. Public Health. 2004;118:403–8. 10.1016/j.puhe.2004.05.010 15313593

[11] Chen KT, Chen WJ, Malilay J, Twu SJ. The public health response to the Chi-Chi earthquake in Taiwan, 1999. Public Health Rep. 2003;118:493–9. 14563906 10.1093/phr/118.6.493 PMC1497595

[12] Woersching JC, Snyder AE. Earthquakes in El Salvador: a descriptive study of health concerns in a rural community and the clinical implications—part II. Disaster Manag Response. 2004;2:10–3. 10.1016/j.dmr.2003.12.007 14760288

[13] Woersching JC, Snyder AE. Earthquakes in El Salvador: a descriptive study of health concerns in a rural community and the clinical implications—part I. Disaster Manag Response. 2003;1:105–9. 10.1016/S1540-2487(03)00049-X 14666095

[14] Malilay J, Real MG, Ramirez Vanegas A, Noji E, Sinks T. Public health surveillance after a volcanic eruption: lessons from Cerro Negro, Nicaragua, 1992. [Medline]. Bull Pan Am Health Organ. 1996;30:218–26. 8897722

[15] Matsuoka T, Yoshioka T, Oda J, Tanaka H, Kuwagata Y, Sugimoto H, The impact of a catastrophic earthquake on morbidity rates for various illnesses. Public Health. 2000;114:249–53. 10.1016/S0033-3506(00)00339-5 10962585

[16] Tanaka H, Oda J, Iwai A, Kuwagata Y, Matsuoka T, Takaoka M, Morbidity and mortality of hospitalized patients after the 1995 Hanshin-Awaji earthquake. Am J Emerg Med. 1999;17:186–91. 10.1016/S0735-6757(99)90059-1 10102325

[17] Asari Y, Koido Y, Nakamura K, Yamamoto Y, Ohta M. Analysis of medical needs on day 7 after the tsunami disaster in Papua New Guinea. Prehosp Disaster Med. 2000;15:9–13. 11183459 10.1017/s1049023x00025024

[18] Vahaboglu H, Gundes S, Karadenizli A, Mutlu B, Cetin S, Kolayli F, Transient increase in diarrheal diseases after the devastating earthquake in Kocaeli, Turkey: results of an infectious disease surveillance study. Clin Infect Dis. 2000;31:1386–9. 10.1086/317500 11096007

[19] Bayramgurler D, Bilen N, Namli S, Altinas L, Apaydin R. The effects of 17 August Marmara earthquake on patient admittances to our dermatology department. J Eur Acad Dermatol Venereol. 2002;16:249–52. 10.1046/j.1468-3083.2002.00488.x 12195564

[20] Sencan I, Sahin I, Kaya D, Oksuz S, Yildirim M. Assessment of HAV and HEV seroprevalence in children living in post-earthquake camps from Duzce, Turkey. Eur J Epidemiol. 2004;19:461–5. 10.1023/B:EJEP.0000027357.57403.3a 15233319

[21] Lora-Suarez F, Marin-Vasquez C, Loango N, Gallero M, Torres E, Gonzalez MM, Giardiasis in children living in post-earthquake camps from Armenia (Colombia). BMC Public Health. 2002;2:5. 10.1186/1471-2458-2-5 11914149 PMC101378

[22] Saenz R, Bissel RA, Paniagua F. Post-disaster malaria in Costa Rica. Prehosp Disaster Med. 1995;10:154–60. 10155423 10.1017/s1049023x00041935

[23] Schneider E, Hajjeh RA, Spiegel RA, Jibson RW, Harp EL, Marshall GA, A coccidioidomycosis outbreak following the Northridge, Calif, earthquake. JAMA. 1997;277:904–8. 10.1001/jama.1997.03540350054033 9062329

[24] Centers for Disease Control and Prevention. Surveillance in evacuation camps after the eruption of Mt. Pinatubo, Philippines. MMWR Surveill Summ. 1992;41:9–12. Medline Erratum in MMWR Surveill Summ. 1992;41:963. 1528189

[25] Magpantay RL, Abellanosa IP, White ME, Dayrit MM. Measles among Aetas in evacuation centers after volcanic eruption. International Scientific Conference on Mt. Pinatubo; Department of Foreign Affairs, Manila; 1992 May 27–31. p. 33.

[26] Banzon Bautista C. The Mount Pinatubo disaster and the people of central Luzon [monograph on the Internet]. 1999 Jun 10 [cited 2006 Feb 15]. Available from http://pubs.usgs.gov/pinatubo/cbautist

[27] Logue JN, Evans Melick M, Hansen H. Research issues and directions in the epidemiology of health effects of disasters. Epidemiol Rev. 1981;3:140–62. 7030759 10.1093/oxfordjournals.epirev.a036231

[28] Sack RB, Siddique AK, Longini IM, Nizam A, Yunus M, Islam MS, 4-year study of the epidemiology of *Vibrio cholerae* in four rural areas of Bangladesh. J Infect Dis. 2003;187:96–101. 10.1086/345865 12508151

[29] Briët O, Galappaththy G, Konradsen F, Amerasinghe P, Amerasinghe F. Maps of the Sri Lanka malaria situation preceding the tsunami and key aspects to be considered in the emergency phase and beyond. Malar J. 2005;4:8. 10.1186/1475-2875-4-8 15676073 PMC548668

[30] Baxter P, Ancia A. Human health and vulnerability in the Nyriagongo volcano crisis, DR Congo Jun 2002 [monograph on the Internet]. 2002 Jun [cited 2006 Feb 15]. Available from http://www.reliefweb.int/rw/rwb.nsf/AllDocsByUNID/302be587c8df7c39c1256be2002cf5cc

[31] Thieren M, Guitteau R. Identifying cadavers following disasters: why? Disasters. 2000;80:1–2.

[32] Morgan O. Infectious disease risks from dead bodies following natural disasters. Rev Panam Salud Publica. 2004;15:307–12. 10.1590/S1020-49892004000500004 15231077

[33] Piarroux R. Cholera: epidemiology and transmission. Experience from several humanitarian interventions in Africa, Indian Ocean and Central America [article in French]. Bull Soc Pathol Exot. 2002;95:345–50. 12696373

